# Micropropagation and Cryopreservation of Yukon Draba (*Draba yukonensis*), a Special Concern Plant Species Endemic to Yukon Territory, Canada

**DOI:** 10.3390/plants10102093

**Published:** 2021-10-03

**Authors:** Akansha Saxena, Wen-Lu Bi, Mukund R. Shukla, Syd Cannings, Bruce Bennett, Praveen K. Saxena

**Affiliations:** 1 Gosling Research Institute for Plant Preservation, Department of Plant Agriculture, University of Guelph, Guelph, ON N1G 2W1, Canada; asaxen01@uoguelph.ca (A.S.); wenlubi@uoguelph.ca (W.-L.B.); mshukla@uoguelph.ca (M.R.S.); 2 Environment and Climate Change Canada, 91780 Alaska Highway, 1st Floor, Whitehorse, YK Y1A 5X7, Canada; Syd.Cannings@ec.gc.ca; 3 B.A. Bennett Herbarium (BABY), 33 Chinook Lane, Whitehorse, YT Y1A 5Y2, Canada; brbennett@klondiker.com

**Keywords:** conservation, cryobanking, droplet-vitrification, in vitro shoot tips, in vitro technologies, Yukon Draba

## Abstract

Yukon Draba (*Draba yukonensis*) is a small, short-lived perennial mustard species that is endemic to southwestern Yukon in Canada. This plant has been categorized as a species of Special Concern. It faces the threat of habitat loss due to natural and man-made causes and a population that is unevenly distributed to a few large and several small subpopulations in the area. It will therefore be judicious to undertake investigations on the conservation of this species to save it from further deterioration which may lead to its extinction. In this study, a protocol was developed for in vitro propagation and cryopreservation of Yukon Draba. The micropropagation protocol was optimized using shoot tips which enabled clonal propagation and in vitro storage of the species. Shoots grew best in the medium containing MS basal salts and had the highest multiplication with the addition of 2 µM 6-benzylaminopurine or 5 µM Kinetin with 3% sucrose. The addition of 10 µM Indole Butyric Acid (IBA) produced the highest number of adventitious roots on the shoots and the longest root length was observed at 2 µM IBA. The rooted plantlets were transferred to greenhouse and the highest survival (87.5%) was observed for the plantlets treated with a lower concentration of IBA (2 µM). Cryopreservation protocol was developed using the droplet-vitrification method for in vitro shoot tips. Two-week-old shoots had the highest survival and regrowth following exposure to plant vitrification solution 3 (PVS3) for 30 min, prior to direct immersion of the droplets into the liquid nitrogen. The optimized protocols for the micropropagation and cryopreservation may be useful for the long-term germplasm conservation and reintroduction of this species in its natural habitat.

## 1. Introduction

Yukon Draba (*Draba yukonensis* A.E. Porsild) is a small, short-lived, perennial species of mustard (family Brassicaceae) with untoothed leaves covered with distinctive stiff and unforked hairs. This species is endemic to southwestern Yukon, Canada. The species is currently located in 19 sites over an area of 477,295 km^2^ [1]. The Yukon Draba population is estimated between 160,200 to 333,000 individuals [1]. The plant survives extreme cold and dry weather conditions and requires full sun and well-drained soils. However, the physiology of the life cycle of this plant is understudied and the mechanisms of cold tolerance remain unknown. Yukon Draba can disperse via seeds, but this ability is apparently limited, with no known method of long-distance dispersal, and an inability to colonize nearby apparently suitable habitat.

The species’ greatest threat is habitat loss as the habitat shifts from open meadows to shrubs and forests, which has been accelerated by the effects of climate change [1]. The habitat that remains is being degraded by several factors, including invasion of grasslands by exotic plants [2], unknown effects of bison disturbance, human intrusions and disturbance, severe weather, and climate change. Furthermore, the uneven distribution of small subpopulations in the area is another factor that restricts the wide dispersal of Yukon Draba. The mature individuals have been reported to be declining in the qualitative assessment of vegetation succession at Alsek meadow along with a decline in the area of the habitat [1]. In view of such threats, Yukon Draba has been assessed as a species of “Special Concern” in 2018 [1]. NatureServe considers Yukon Draba to be globally Imperiled to Vulnerable (G2G3). It is possible that if no measures are taken, this plant may become threatened and eventually become extinct. This necessitates the development of effective strategies for the long-term conservation of this species.

Micropropagation is a useful technique for the conservation of endangered plants through mass propagation [3,4,5,6,7,8]. The application of micropropagation with conservation priority has saved several economically important plant species from extinction. Grigoriadou et al. [9] reported that 22 native Greek high value medicinal and aromatic plant species such as *Achillea occulta, Amsonia orientalis, Anthyllis splendens,* and *Calamintha cretica* were successfully micropropagated and acclimatized. African Cherry (*Prunus Africana*), an endangered medicinal tree and *Magnolia sirindhorniae,* an endangered ornamental species, were also successfully micropropagated and recommended for large scale production [10,11]. Such studies demonstrate the usefulness of micropropagation in the reintroduction of endangered species as well as potential improvement of breeding programs by incorporating the wild relatives of commonly cultivated crops which are an important source of agronomically important genes. For example, Himalayan Chickpea (*Cicer microphyllum*), an endemic wild relative of chickpea (*Cicer arietinum*), has been successfully micropropagated and used in conservation and breeding programs [12]. Thus, micropropagation techniques offer significant help in the revival of endangered plant species and as such, the technique may also be applied to plants of commercial importance [5,13].

Cryopreservation of plant tissues allows for long-term storage and subsequent regeneration of endangered germplasm [14,15,16,17,18,19]. Plant tissues are stored within cryo-tanks in either liquid nitrogen (LN, −196 °C) or its vapor phase (LNV, approx. −165 to −190 °C) [20,21,22]. Under cryopreserved conditions, plant tissues are stored in a state in which cellular divisions and metabolic activities are minimal, thus preserving the genetic integrity for a longer duration [16,23,24]. The cryoprotectants are the antifreezing substances used to protect cellular structure; however, their concentration, exposure duration, and temperature need to be optimized for each plant species [25]. The cryopreservation technique ensures long term germplasm conservation and can be used for cryobanking for future reintroductions in natural habitats [15]. Cryopreservation has been successfully utilized for the preservation of critically endangered and endemic species such as Pearl-like Androcalva (*Androcalva perlaria*) [26], Sibiriskt Stenbär (*Rubus humulifolius*) [27], and *Aster altaicus* [28]. Recently, Streambank lupine (*Lupinus rivularis*), an endangered plant in Canada, has also been micropropagated and cryopreserved successfully [29]. The cryopreserved explants can be stored as a source of true-to-the-type germplasm for use in breeding programs of economically important crops [30,31,32,33].

The Yukon Draba’s rarity, small distribution in a habitat that is declining in quality, and the longer duration of established plants to flowers makes this species a worthy candidate for in vitro conservation. This approach will help to rapidly multiply the plants and store germplasm for long-term conservation. To date, micropropagation of Yukon Draba has not been reported. The objectives of the current study were to develop efficient protocols for in vitro mass propagation and cryopreservation of Yukon Draba for long term storage of the germplasm and potential reintroduction in its natural habitat.

## 2. Results

### 2.1. Micropropagation

#### 2.1.1. Culture Initiation

**Figure 1:**
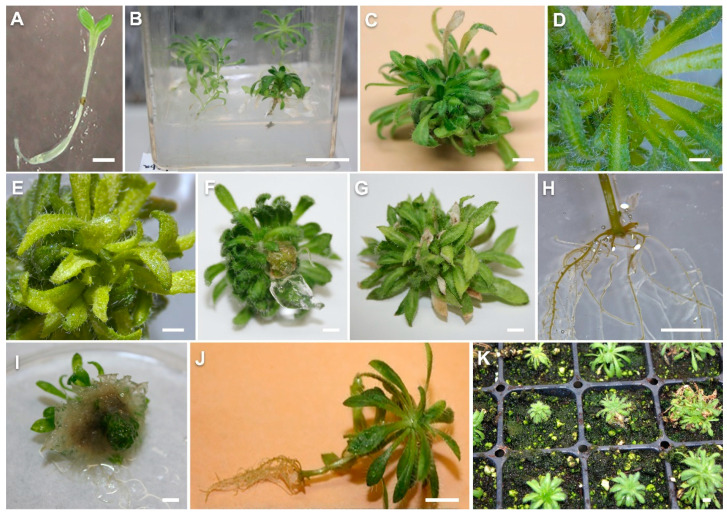
Yukon Draba seed germinated on MS basal medium (**A**). Shoot cultures maintained on the MS medium supplemented 2.2 μM BA, 1μM GA_3_ and 3% sucrose (**B**) for 6 weeks in culture (**C**). The leaves on the shoots cultured on MS media had more leaf hair and were greener (**D**) and the leaves on the shoots were cultured on SH medium (**E**). In vitro shoot growth on MS basal medium (**F**) and MS medium supplemented 5.0 μM BA (**G**) after 4 weeks of culture. Distinct in vitro rooting response was observed when shoots were cultured on the MS medium supplemented 5.0 μM IBA (**H**) and 2.0 μM NAA (**I**). In vitro shoots developed on the MS rooting medium supplemented 5.0 μM IBA (**J**) and plants transferred to the greenhouse for acclimatization (**K**). Bar in (**A**) = 1 mm, bars in (**B**–**K**) = 1 cm.

The seeds of Yukon Draba germinated in five days and developed cotyledonary leaves in eight days (Figure 1A). The emergence of rosette leaves from seedlings started in 10 days. At the end of 20 days, seedlings had 4–6 rosette leaves. The seed germination percentage was observed between 23–53% for different seed populations collected from different plants. One-month-old seedlings transferred to maintenance medium showed continued growth (Figure 1B).

#### 2.1.2. Shoot Proliferation: Basal Salts

**Figure 2:**
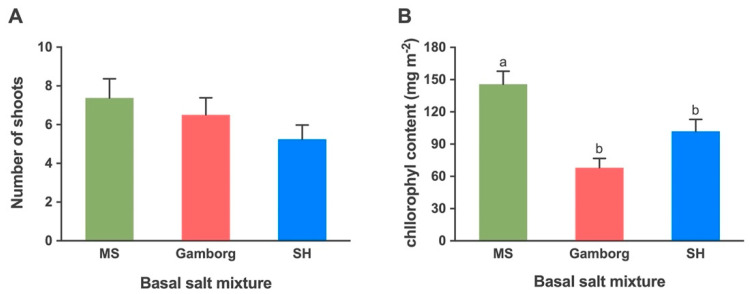
The effects of basal salts of three tissue culture media (MS, Gamborg and SH) on (**A**) number of shoots and (**B**) average chlorophyll content (mg m^−2^). Bars represent means ± standard error, where means followed by the different letters are significantly different at *P* ≤ 0.05 according to Tukey–Kramer HSD test. Each level consisted of ten biological replicates.

Three common basal salt mixtures of the MS, SH and Gamborg media were tested at full strength for shoot proliferation. The number of shoots produced in these three different basal media was 7.4, 5.3 and 6.5, respectively, which were not different significantly (Figure 2A). The leaves on MS medium were greener with more leaf hairs (Figure 1C,D) compared to the leaves on SH (Figure 1E). Purple black dots were also observed on upper leaves in shoots on all the treatments but slightly lesser on MS medium as compared to SH and Gamborg media. The leaves produced on the Gamborg medium were narrower and less hairy as compared to other treatments. Yellowing of older leaves was observed in all cultures regardless of the medium composition.

The average chlorophyll content measured in leaves exhibited a significantly higher average in shoots cultured on MS medium compared to SH and Gamborg media. The average chlorophyll content (mg m^−2^) in shoots cultured on MS, SH and Gamborg was 145.7, 101.9 and 68.0, respectively (Figure 2B).

#### 2.1.3. Effect of Cytokinin on Shoot Multiplication

**Figure 3:**
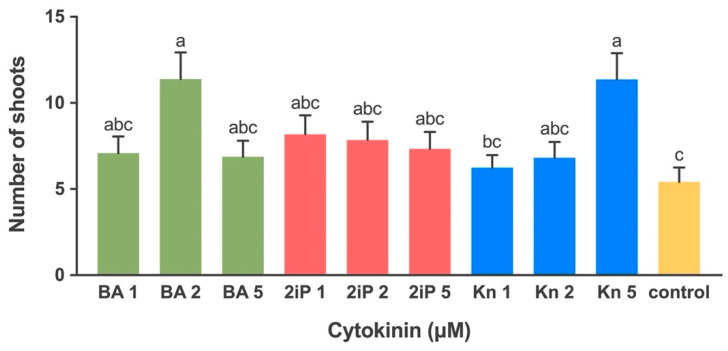
The effects of three cytokinins, 6-benzylaminopurine (BA), 2-isopentenyladenine (2iP), and kinetin (Kn) (0, 1, 2, 5 μM) on numbers of shoots after 4 weeks of culture. Bars represent means ± standard error, where means followed by the different letters are significantly different at *P* < 0.05 according to Tukey–Kramer HSD test. Each level of the cytokinins tested consisted of ten biological replicates.

The numbers of shoots produced was affected by different types and levels of cytokinins (Figure 3). The highest number (11.4) of shoots was recorded at 5 µM Kn and 2 µM of BA (Figure 1G) which were significantly different from control (Figure 1F) and Kn at 1 µM. The number of shoots produced on the media with BA at 1 µM and 5 µM was 7.1 and 6.9, respectively. No significant difference was observed in shoot production induced at different levels of 2iP. The mean number of shoots produced at 1, 2, 5 µM of 2iP was 8.2, 7.9 and 7.3, respectively. The mean number of shoots produced by Kn at 1 and 2 µM was 6.2 and 6.8, respectively (Figure 3). The shoots produced on the media supplemented with Kn and BA were normal while those grown on 2iP enriched media were hyperhydric with a glassy appearance.

#### 2.1.4. In Vitro Rooting

**Figure 4:**
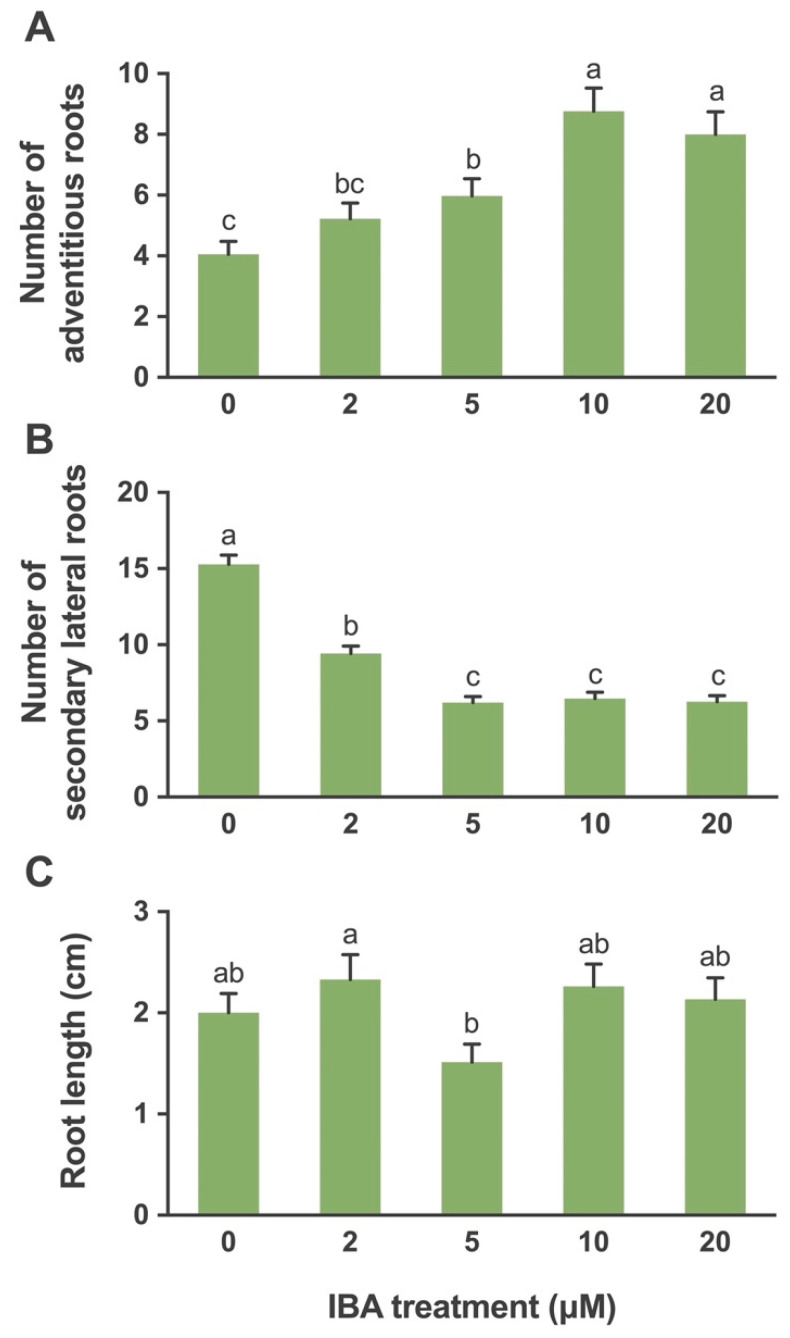
The effects of auxin IBA (0, 2, 5, 10, 20 μM) on (**A**) number of adventitious roots (**B**) number of secondary lateral roots and (**C**) root length developed on shoot after 4 weeks of culture. Bars represent means ± standard error, where means followed by the different letters are significantly different at *P* ≤ 0.05 according to Tukey–Kramer HSD test. Each level of the auxin tested consisted of ten biological replicates.

The root formation responses varied with the type and concentration of auxin added in the medium. In the case of IBA, there was no callusing at the crown region (Figure 1H). The adventitious roots emerged from the crown region of the plant and distinct secondary lateral roots emerged from these adventitious roots. However, in the case of NAA, callus formation at the base of the crown was observed. Small hairy roots with no branches developed from the crown region (Figure 1I). The level of IBA affected the number of adventitious roots produced with significantly greater response at IBA levels of 10 and 20 µM as compared to the control and other IBA levels (Figure 1J). The mean number of adventitious roots produced at 0, 2, 5, 10, and 20 μM was 4.1, 5.2, 6.0, 8.8, and 8.0, respectively (Figure 4A).

The number of secondary lateral roots was affected by the IBA levels. The control produced a significantly higher number of secondary lateral roots. No significant differences were observed among the levels of IBA at 5, 10 and 20 μM. The mean numbers of secondary lateral roots produced at 0, 2, 5, 10 and 20 μM were 15.3, 9.4, 6.2, 6.5, and 6.3, respectively (Figure 4B).

The root length was affected by the various IBA levels. A significant difference between root length in treatments with IBA 5 μM and IBA 2 μM was recorded. The IBA at 2 μM gave the best result as compared to the rest of the other treatments. The root lengths at 0, 2, 5, 10 and 20 μM levels of IBA were 2.0, 2.3, 1.5, 2.3, and 2.1 cm, respectively (Figure 4C).

The NAA levels also affected the number of roots produced. The NAA at 10 μM produced a significantly higher number of roots as compared to the control and other levels. The mean number of roots produced at 0, 2, 5, 10 μM levels were 8.6 ± 1.75, 8.6 ± 1.76, 17.5 ± 3.51, and 22.6 ± 4.52, respectively. The root length was not affected by the NAA levels. The mean root length at 0, 2, 5, 10 μM was 0.82 ± 0.23, 0.82 ± 0.11, 1.1 ± 0.1, and 1.2 ± 0.12 cm, respectively.

#### 2.1.5. Greenhouse Acclimatization

The plantlets which developed roots in NAA treatments did not survive in the mist bed. However, the plantlets developed in IBA treatments at 0, 2, 5, 10 μM survived in the greenhouse with mean survival percentages of 87.5%, 87.5%, 77.5%, 85.0%, respectively (Figure 1J,K).

### 2.2. Cryopreservation

#### 2.2.1. Effect of Stock Plant Age on Survival and Regrowth of Cryopreserved Shoot Tips

**Figure 5:**
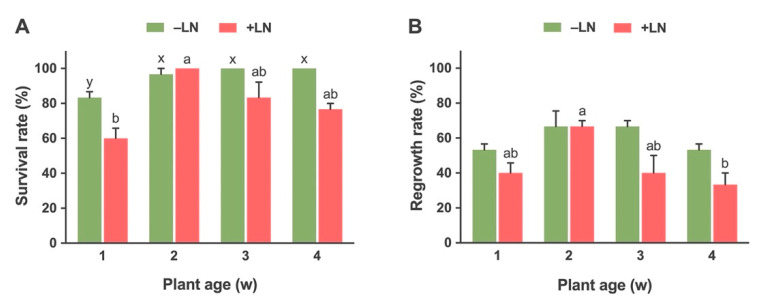
Effects of plant age on survival (**A**) and regrowth (**B**) of the control (−LN) and cryopreserved (+LN) shoot tips of Yukon Draba following droplet-vitrification. Shoot tips were precultured for 1 d with 0.3 M sucrose solution. Precultured shoot tips were treated for 20 min with a loading solution composed of 1.9 M glycerol and 0.5 M sucrose, followed by exposure to plant vitrification solution 3 (PVS3) for 30 min, prior to direct immersion of the droplets into liquid nitrogen. 1, 2, 3 and 4 represent the age of stock plants in a week. Results are presented as means ± SE and different letters indicate significant differences at *P* < 0.05 by Tukey’s range test.

Age of the stock plant that provided explants affected the survival and regrowth rates after cryopreservation (Figure 5A,B). The survival rate for non-cryopreserved (-LN) explants was significantly higher for 2 weeks (96.6%), 3 weeks (100%) and 4 weeks (100%) old stock plants as compared to 1-week-old (83.33%) stock plants. The regrowth percentage of explants was similar for all non-cryopreserved (−LN) plants irrespective of plant age; 53.33%, 66.67%, 66.67%, and 53.33% for 1, 2, 3, and 4-week-old plants, respectively (Figure 5B).

**Figure 6:**
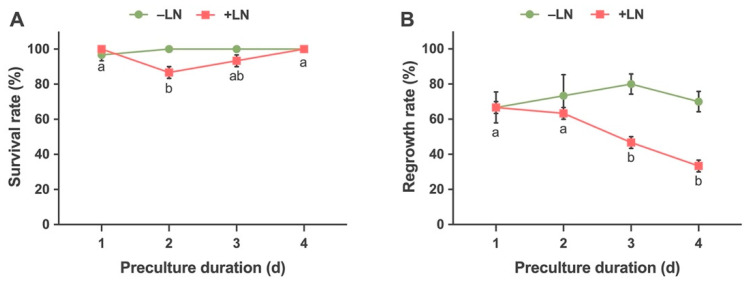
Effects of preculture duration on survival (**A**) and regrowth (**B**) of the treated control (−LN) and cryopreserved (+LN) shoot tips in Yukon Draba following droplet-vitrification. Shoot tips were precultured for 1, 2, 3, 4 d with 0.3 M sucrose solution and precultured shoot tips were treated for 20 min with a loading solution composed of 1.9 M glycerol and 0.5 M sucrose, followed by exposure to plant vitrification solution 3 (PVS3), prior to direct immersion of the droplets into liquid nitrogen. Results are presented as means ± SE and different letters indicate significant differences at *P* < 0.05 by Tukey’s range test.

In the case of cryopreserved shoot tips (+LN), the survival rate was significantly higher in 2-week-old stock plants (100%) as compared to the rest of the treatments. The explants from 3-week (83.33%) and 4-week-old (76.6%) stock plants showed higher survival as compared to 1-week-old plants (60%). The regrowth was significantly higher for a 2-week-old plant (66.67%) as compared to other treatments (Figure 5A,B). The regrowth of explants from 1-week and 3-week-old plants was about 40% while the per cent regrowth of explants from 4-week-old plants was the lowest (33.33%) (Figure 6B).

#### 2.2.2. Effects of Preculture Duration on Survival and Regrowth

The shoot tips were precultured for 1, 2, 3 and 4 days with 0.3 M sucrose solution. The survival and regrowth responses of non-cryopreserved (−LN) shoots were not affected significantly by the duration of preculture (Figure 6A,B). The survival rate was 96.67% for 1 day and 100% for 2, 3 and 4-day treatments. The regrowth rate was 66.67%, 73.33%, 80% and 70%, respectively, for 1, 2, 3 and 4 days of treatment. In the case of cryopreserved shoots tips (+LN), the treatment duration affected both the survival and regrowth responses. The survival rates of 1 and 4-day preculture treatment was significantly higher as compared to other treatments. The survival rate was 100% for 1 and 4-day treatments which was followed by 3-day preculture treatment which had a survival rate of 93.33%. The lowest response (86.67%) was observed for explants from the treatment of 2-day duration.

The regrowth rate was significantly higher in shoot tips treated for 1 and 2-day periods as compared to 3 and 4-day periods. Shoot tips precultured for 1 and 2 days had a regrowth rate of 66.67% and 63.33%, respectively, while the 3 and 4 days precultured shoots had low regrowth rates of 46.67% and 33.33%, respectively (Figure 6A,B).

#### 2.2.3. Effects of Time Durations of Exposure to Vitrification Solution (PVS3) on Survival and Regrowth

**Figure 7:**
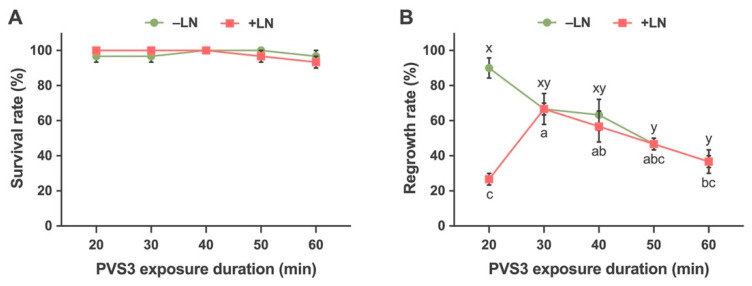
Effects of PVS3 exposure on survival (**A**) and regrowth (**B**) of the treated control (−LN) and cryopreserved (+LN) shoot tips in Yukon Draba following droplet-vitrification. Shoot tips were precultured with 0.3 M sucrose solution. One-day precultured shoot tips were treated for 20 min with a loading solution composed of 1.9 M glycerol and 0.5 M sucrose, followed by exposure to plant vitrification solution 3 (PVS3) for 20, 30, 40, 50, 60 min, prior to direct immersion of the droplets into liquid nitrogen. Results are presented as means ± SE and different letters indicate significant differences at *P* < 0.05 by Tukey’s range test.

The effect of duration of PVS3 treatment had no significant effect on the survival of non-cryopreserved shoot tips (−LN). The PVS3 treatment for 20, 30, 60 min had a survival rate of 96.67% and 40- and 50-min treatments had a survival rate of 100% (Figure 7A). However, the regrowth rate was affected by the duration of the PVS3 treatment. The 20 min treatment was best for regrowth rate and significantly different as compared to others. The PVS3 treatment duration of 50 and 60 min was significantly lower for regrowth rate among all treatments. The regrowth rate for treatment duration of 20,30,40,50 and 60 min were 90, 66.67, 63.33, 46.67 and 36.67%, respectively (Figure 7B).

The duration of PVS3 treatment had no significant effect on the survival rate of cryopreserved shoot tips (+LN). The mean survival rates for 20, 30, 40, 50 and 60 min were 100, 100,100, 96.67 and 93.33%, respectively (Figure 7A). The regrowth rate was significantly affected by the treatment duration. The optimal PVS3 treatment period was determined to be a 30 min exposure, which resulted in an average regrowth of 66.67% (Figure 7B).

#### 2.2.4. Thermal Analysis

**Figure 8:**
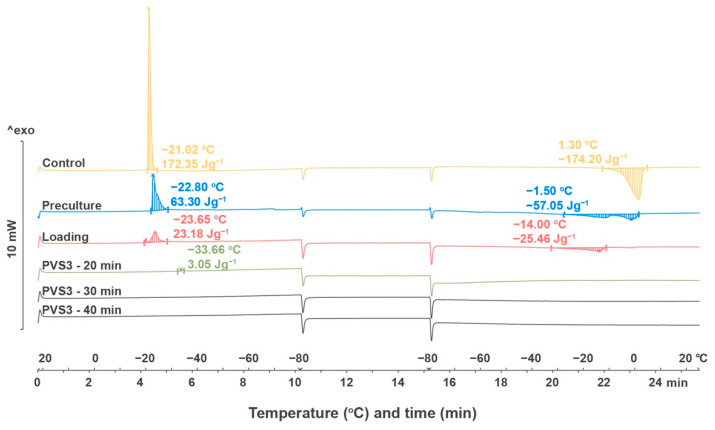
Differential scanning calorimeter (DSC) thermographs of Yukon Draba shoot tips which were freshly dissected, precultured, loaded, and dehydrated with PVS3 for 20, 30, and 40 min.

The thermal behaviors of shoot tips without treatment, after preculture, loading treatment and dehydration were observed during cooling and rewarming processes (Figure 8). The onset temperature for cooling dropped from −21.02 °C in control shoot tips to −22.80 °C in precultured shoot tips, to −23.65 °C in shoot tips treated with loading solution, and to −33.66 °C in shoot tips dehydrated with PVS3 for 20 min. The onset temperature for melting dropped from 1.30 °C in the control shoot tips to −1.50 °C in precultured shoot tips, and to −14.0 °C in shoot tips treated with the loading solution. Areas of the melting transition were decreased for the shoot tip samples from control (172.35 Jg^−1^) to preculture (63.30 Jg^−1^), to loading solutions (23.18 Jg^−1^), and to dehydration with PVS3 for 20 min (3.05 Jg^−1^). There was no peak observed during the freezing and rewarming process when shoot tips were dehydrated with PVS3 for 30 min, 40 min (Figure 7), 50 min, and 60 min (Data not shown).

## 3. Discussion

The objective of this study was to develop in vitro techniques for the conservation of Yukon Draba, a plant species of Special Concern. Another reason for developing micropropagation and cryopreservation techniques for this species was its endemic nature and the populations being restricted to extremely cold environments in the southwestern Yukon. The strength of the micropropagation protocol lies in the fact that it needs very little starting material to initiate the cultures for producing multiple plants in a short time frame. This study implements the in vitro technique for plant conservation and provides a model for in vitro conservation and revival of Yukon Draba (Figure 1). Additionally, the plant grows in extreme cold conditions and can be used to study the molecular and physiological mechanism of cold tolerance similar to the studies in Mouse-ear Cress (*Arabidopsis thaliana*) and Saltwater Cress (*Eutrema salsugineum*) [34]. The reintroduction of a threatened species in its natural habitat is expected to expedite future efforts in species restoration projects.

The seed germination and growth of the seedlings of endangered plant species are limited due to several physical, physiological, and biochemical factors [8]. However, the Yukon Draba seeds germinated in five days on MS basal medium. The seeds displayed no signs of physical, physiological, and chemical dormancy. The ease of germination of Yukon Draba seeds with a moderate seed germination percentage is an important contributing factor in the successful development of the micropropagation protocol.

Yukon Draba shoots cultured on MS basal salt and vitamins (BM) medium without cytokinin, and GA_3_ turned white and died. Therefore, the 20-day-old shoots were transferred to an MS medium containing 1 µM GA_3_ and 2.2 µM BA (SMM). Plant growth regulators have been commonly used for successful cryopreservation of shoot tips and play a key role in obtaining a higher shoot regrowth after cryopreservation [22].

Results showed that there was no effect of the type of basal media evaluated for a number of shoots and average chlorophyll content. There was no significant variation in the number of shoots produced on MS, SH and Gamborg media. However, significantly a higher number of shoots was produced on the MS medium. The yellowing of leaves and the appearance of purple-black dots were observed in all treatments, which might be due to plant age or an imbalance of micronutrients such as manganese or zinc [35,36]. The leaves on MS medium were hairier and greener as compared to other basal media. The average chlorophyll content of the shoots cultured on MS media was also significantly higher as compared to other basal media suggesting that the MS composition provides an optimum balance of salts in the medium. The three basal media tested vary in the source of nitrogen and quantity of zinc sulphate, calcium chloride and manganese sulphate. The MS medium has the highest quantity of nitrogen source, Zinc Sulfate•7H_2_O, Manganese Sulfate•H_2_O and Calcium Chloride which can be a reason for greener leaves, more leaf hair and higher chlorophyll content. Similar results were observed for Cherry Birch (*Betula lenta*) as shoots cultured on Driver and Kuniyuki Walnut medium (DKW) [37] and MS media performed better than those on the Woody Plant Medium (WPM) due to the reduced amount of ammonium nitrate present in WPM as compared to the other two [38]. In highbush blueberry (*Vaccinium* spp.) the best medium for shoot optimization was found to be a mixture of equal parts of MS and WPM as compared to MS or WPM [39]. The shoots cultured on MS medium were hyperhydric due to ammonium ions and WPM contained neither cobalt nor iodine; thus, the combination of the two media was complementary. However, in the micropropagation of Cardamom (*Elettaria cardamomum*), the SH media was found to be the best [40]. The highest average chlorophyll content was found in Yukon Draba plants cultured on the MS medium compared to others, probably due to differences in micronutrient levels. Similar results were reported in Candyleaf (*Stevia rebaudiana*) where plants regenerated on higher levels of micronutrients showed a significant increase in leaf chlorophyll content [41]. Similarly, the media containing WPM and Anderson’s Rhododendron Medium (ARM) basal salts with vitamins favorably affected the growth and development of the explants and prevented the occurrence of hyperhydricity and premature senescence of in vitro propagated plantlets of Cherry Plum, *(Prunus cerasifera*) [42]. The requirement of basal medium depends on the genotype, culture conditions, and culture duration. The development of customized basal media with optimized mineral composition may be more effective for micropropagation compared to the use of basal media established for other species [43,44]. For example, a systematic approach for determining the mineral nutrient factors has been developed to improve hazelnut shoot growth and development under in vitro conditions [43,45].

Cytokinins are important for plant cell division, growth, morphogenesis, and development [46]. Skoog and Miller [47] demonstrated that the ratio of auxin and cytokinin in the medium determined the shoot and root production in tobacco cultures with a high cytokinin: auxin leading to shoot development, whereas the opposite promoting the root formation. In Yukon Draba, the highest numbers of normal shoots were produced with 2 µM BA and 5 µM Kn, while the shoots produced with 2-IP were hyperhydric. Similarly, BA was found effective for several endangered plant species such as Hill’s Thistle (*Cirsium hillii*) [48], Golden Paintbrush (*Castilleja levisecta*) [19], Cherry Birch [38], and Estonian Snow Lotus (*Saussurea esthonica*) [49]. Kinetin has also been used for shoot proliferation in various plants such as *Limoniastrum monopetalum* [50], *Cucumis sativus* [51], and *Dioscorea alata* [52]. These studies indicated that the tissue culture response to cytokinin was species-dependent.

Hyperhydricity in plants of tissue culture origin is a common physiological disorder that gives the plants a glassy appearance. The main causes of hyperhydricity in tissue culture-grown plants are oxidative stresses, gas accumulation in the vessel, duration between subcultures as well as the number of subcultures, concentration and type of gelling agent, the type of explants used, and the level of nutrients and hormones [53]. Studies have shown that cytokinins could induce hyperhydricity during in vitro propagation [54,55]. The shoots of Yukon Draba produced on 2-IP media were hyperhydric. Similar results were observed in *Aloe polyphylla* shoots produced on BA supplemented medium [56]. In Pear (*Pyrus pyrifolia*), the use of thidiazuron (TDZ) and N-(2-chloro-4-pyridyl)-N′-phenylurea (CPPU) led to higher hyperhydricity of cultures than BA and kinetin [55]. It is well documented that high levels of cytokinins lead to hyperhydricity as compared to low levels but how cytokinins induce hyperhydricity remains to be investigated [57,58]. Cytokinin is known to cause fasciated stems, breakable leaves, and reduced chlorophyll accumulation and various stress like symptoms in plants [59,60,61].

The auxins have several functions in plants such as the control of cell division, elongation, as well as the differentiation of cells and adventitious root formation [62]. In the case of Yukon Draba, IBA treated plants produced adventitious roots from the crown of the plant and further distinct secondary lateral roots emerged from these adventitious roots. In the NAA treated shoots callus formation was observed at the base of the crown and hairy roots with no clear distinction between primary and secondary roots developed from the crown region. The highest numbers of adventitious roots were produced with 10 μM IBA and the maximum numbers of mean secondary lateral roots were produced in the control treatment. The formation of the primary adventitious roots increased with the level of auxin and a reverse trend was observed in the production of secondary lateral roots. The maximum root length was observed at 2 μM IBA as compared to all other treatments. IBA has been found to be effective for inducing root development in endangered plant species such as Bredasdorp Conebush (*Leucadendron laxum*) [63]. Air layers of the endangered Azorean tree, Pau-branco (*Picconia azorica*), produced maximum roots with IBA treatment [64]. IBA is commonly used for promoting root formation as it is non-toxic at variable concentrations and is efficient in stimulating the rooting of shrubs and evergreens [65]. However, endangered plant species have also been rooted successfully with NAA alone or in combination with IBA [48,66]. These studies indicate that tissue response to auxin depends on the type and concentration of the auxin used and the physiological status of the explant and is genotype-specific [67,68]. An additional factor in root initiation is the interaction between endogenous and exogenous auxin [69] which can influence the transplant and survival of plantlets. In the greenhouse, the plants cultured on NAA were not able to survive which may be due to the callus development at the crown. The highest survival in the greenhouse was observed with Yukon Draba plantlets developed on lower levels of IBA. This might be related to a higher number of secondary lateral roots in these plantlets. Lateral roots help in increased water and nutrient uptake; they also help in securing the plant into the soil and increase the surface area of the root system [70].

Cryopreservation is a valuable tool for the conservation and long-term storage of endangered plant species [8,29,71,72]. Commonly used explants such as meristems, nodes, buds, roots and seeds can be used for the plant species with irregular seed production and the species in which seed collection is limited due to accessibility [72]. Since 2002, the Committee on the Status of Endangered Wildlife in Canada (COSEWIC) has recognized several plant species as Endangered, Threatened, or Special Concern in Canada. One such species is Yukon Draba, which is habitat-specific as it is located in 19 sites in the Yukon Territory. Due to a lack of starting seed material, seeds were not used for cryopreservation. Instead, shoot tips from in vitro propagated plants were used to develop the cryopreservation protocol. Shoot tips have been commonly used as explants for cryopreservation studies in several species such as Golden Paintbrush [19], Cherry Birch [38], Hemp (*Cannabis sativa*) [73], and the German Großes Sandröschen (*Tuberaria major*) [74].

The plant age and physiological status of the starting plant material are important factors for successful shoot tip cryopreservation [14,21,22,75]. The age of source plants that provide explants may determine the competence of explants to withstand various treatments of cryopreservation and subsequent regrowth [76]. Our results showed that the use of 2-week-old in vitro stock cultures resulted in higher recovery after cryopreservation as compared to 1, 3 and 4-week-old plants. Similar findings were reported for Golden Paintbrush plants [19]. In the case of cryopreservation of Sibiriskt Stenbär, the highest survival and regrowth were obtained from 1-month-old buds as compared to 2 and 4-month-old buds [27]. Contrasting results were reported in the cryopreservation of Apple (*Malus domestica*), cv. pinova [77], Grapevine (*Vitis vinifera*) [78] and Plymouth Pear (*Pyrus cordata*) [79] which showed higher recovery with older shoots. However, the age of the explants had no significant effect on survival and regrowth after cryopreservation for Sweet Potato (*Ipomoea batatas*) [80], and Hill’s thistle [15]. In general, the younger source materials has more vigour due to higher numbers and activity of meristematic cells which may support better recovery from the cryopreservation treatments [19]. Additionally, the use of younger explant sources can reduce the time and resources to maintain cultures for experimentation.

The preculture treatment provides osmotic dehydration of the plant material by application of sugars, sugar alcohols and polyethylene glycol (PEG) [81]. In our study, we used a 0.3 M sucrose solution for preculture. The duration of preculture was tested for 1, 2, 3 and 4 days. The regrowth was best in 1 and 2-day-treated explants as compared to 3 and 4 days of treatment. Similar results were observed in shoot tips of Cherry Birch preculture with 0.3 M sucrose for 24 h [38]. In a study conducted with *Centaurium rigualii*, the regrowth of cryopreserved shoot tips precultured with 0.3 M sucrose for 24 h was significantly better than 0.1 M for 72 h [82]. A 3-day preculture treatment of 0.4 M sorbitol in Dwarf Green Kangaroo Paw (*Anigozanthos viridis*) was optimum. An increasing trend from 1 to 3 days and a declining trend from 3 to 7 days were observed in the survival of shoot apices after cryopreservation [83]. These studies indicate that preculture with a high sucrose concentration for an optimum duration is important for successful cryopreservation. The dehydration tolerance/sensitivity of species and the size of explants are major factors that determine the duration of preculture [84]. Optimization of component concentrations and preculture duration is necessary for enhancing shoot tip tolerance during the dehydration process. However, the influence of these factors varies across crops and cryopreservation methods [22,85,86,87,88,89].

Vitrification based cryopreservation techniques use highly concentrated solutions to remove most or all of the freezable water from the cells through an osmotic process. Vitrification protocols often make use of PVS2 [90] or PVS3 [91]. Cryoprotectant solution exposure time and temperature need to be optimized as tissue sensitivity to PVS2 or PVS3 are species-specific [22,92]. In our study, Yukon Draba shoot tips tolerated a longer (50 min) period of PVS3 exposure at room temperature, but a shorter incubation time (30 min) was sufficient for an adequate level of shoot tip regrowth (66.67%) after LN exposure. Similar results were obtained in Jerusalem Artichoke (*Helianthus tuberous*) shoot tips where the optimal PVS2 exposure was between 15–40 min and the regrowth was 83% [93]. The regrowth of lily (*Lilium × siberia* cv. Siberia) shoot tips was observed to be highest at 90 min PVS2 exposure compared to longer exposure treatments [94]. Grapevine cv. Portan had the highest regrowth (50%) after 50 min PVS2 exposure at 0 °C [95]. Using the same vitrification technique, Bettoni et al. [87] found that shoot tips derived from growth chamber grown plants could not tolerate PVS2 incubation durations that were as long as those used for shoot tips derived from in vitro stock cultures [96]. The optimal PVS2 exposure duration was 30 min for shoot tips derived from growth chamber grown plants and 90 min for those of in vitro cultures. It is likely that in vitro plants were more hydrated and needed longer PVS2 exposures to remove the freezable water. The shoot tips of Flat-leaved Vanilla (*Vanilla planifolia*) could not tolerate longer treatment (60 min) of both PVS2 and PVS3 during cryopreservation [97]. However, contrasting results are observed in some species where the longer duration of PVS2 and PVS3 treatment are optimum. In Bleeding Heart (*Lamprocapnos spectabilis*), shoot tips recovered best after 150-min PVS3 treatment [98]. The shoot tips of Golden Paintbrush had a high regrowth at 100 min of treatment with concentrated PVS3 [19]. Similar results were reported in Cherry Birch [38]. Such studies suggest that certain species are more tolerant to vitrification solution than others and the recovery from vitrification treatment depends on tissue sensitivity, cell viability, and cytotoxicity of the vitrification solution [95,99].

## 4. Materials and Methods

### 4.1. Micropropagation

#### 4.1.1. Seed Germination and Culture Initiation

Seeds of Yukon Draba were collected in collaboration with Yukon Conservation Data Centre and stored at 4 °C. The seeds were surface sterilized with 4% bleach solution (commercial bleach with 5% sodium hypochlorite) for 30 min and rinsed for 30 min with deionized distilled water. Seeds were then treated with 15% bleach solution for 30 min and rinsed three times, 5 min each, in deionized distilled water. Surface sterilized seeds were plated on semi-solid Murashige and Skoog (MS) [100] medium with vitamins (Phytotechnology Laboratories^®^, Shawnee Mission, KS, USA) containing 3% sucrose and 2.2 gL^−1^ phytagel (basal medium—BM) (Sigma-Aldrich, Oakville, ON, Canada). The pH of the medium was adjusted to 5.7 prior to autoclaving at 121 °C and 118 kPa for 20 min. All Petri dishes were kept in the dark for 7 days at 23 ± 2 °C and moved to the growth chamber at 23 ± 2 °C under a 16 h photoperiod at a light intensity of 35 µmol m^−2^ s^−1^ (Osram Sylvania Ltd., Mississauga, ON, Canada).

One-month-old seedlings were transferred to Magenta GA7 vessels (Sigma Aldrich, Oakville, ON, Canada) containing the maintenance medium consisting of the BM supplemented with 1µM gibberellic Acid (GA_3_) and 2.2 µM 6-benzylaminopurine (BA) (Phytotechnology Laboratories) to establish in vitro cultures. Cultures originating from each seed were maintained separately. Uniform shoots that appeared healthy and green were used to optimize the micropropagation protocol, including root initiation and plantlet development; all pale-white, unhealthy shoots were discarded.

#### 4.1.2. Growth Conditions

In each shelf in the tissue culture growth room, two centered fluorescent bulbs (Osram Sylvania Ltd., Mississauga, ON, Canada) were fitted 30 cm above the culture vessels to provide 35 µmolm^−2^ s^−1^ of light with a 16-h photoperiod. The range of temperature in the growth room was 23 ± 2 °C during the day, which decreased up to 5 °C in the dark. A completely randomized design (CRD) was followed in arranging the Petri dishes in the in vitro experiments.

#### 4.1.3. Shoot Multiplication

The effects of three cytokinins were evaluated to optimize in vitro shoot multiplication using shoot tips as explants. Shoots were multiplied from cultures as mentioned above and transferred to media supplemented with 6-benzylaminopurine (BA), 2-isopentenyladenine (2iP), and kinetin (Kn) at 0, 1, 2, 5 µM. The highest concentration was selected from preliminary experiments using BA. The medium consisted of MS basal salts and vitamins, 3% *w/v* sucrose, 1 µM GA_3_, 2.2 gL^−1^ Phytagel (Sigma-Aldrich, Oakville, ON, Canada) and the pH was adjusted to 5.7.

Three commonly used basal salt mixtures MS [100], Schenk and Hildebrandt (SH) [101], and Gamborg [102] were tested to determine the optimal macro- and micronutrients necessary for optimum shoot proliferation. Observations were recorded for the number of shoots and average chlorophyll content in the micropropagated shoots after 4 weeks. All media contained 3% sucrose, MS vitamins, 2.2 μM BA and 1.0 μM GA_3_, and 2.2 gL^−1^ Phytagel adjusted to pH 5.7. The chlorophyll contents were measured using a CCM-300 Chlorophyll Content Meter (Opti-Sciences^®^ Inc, Hudson, NH, USA) for each treatment as an indicator of shoot health. Five leaves were collected per treatment for estimating the chlorophyll content (mg m^−2^). The average obtained from the shoots under each treatment represented the average chlorophyll content for the treatment.

#### 4.1.4. In Vitro Rooting

The effects of two auxins, naphthaleneacetic acid (NAA) and indole butyric acid (IBA), were tested at various levels (0, 2, 5, 10 or 20 μM) using in vitro shoots to optimize in vitro rooting. In vitro shoots were collected from 4-week-old shoot cultures and placed in Magenta GA7 vessels (Sigma Aldrich, Oakville, ON, Canada). Auxins (IBA and NAA) were added to the BM medium supplemented with 1 µM GA_3_, 2.2 gL^−1^ Phytagel and pH adjusted to 5.7. The number of primary adventitious roots (roots emerging from crown of the plant) and secondary lateral roots (roots emerging from primary adventitious roots), and root length were recorded after forty days of culture.

#### 4.1.5. Greenhouse Acclimatization

Plantlets were rinsed with deionized water to remove any excess medium and then transferred to 18 cells trays containing soil mix (Sunshine^®^ Mix #4; Sun Gro Horticulture Canada Ltd., Brantford, ON, Canada). Trays were placed in the mist bed (80% relative humidity), sprayed with water for 15 s every 35 min during the day or every 4 h at night for 2 weeks and later transferred to greenhouse benches (at 250 μmolm^−2^ s^−1^ light intensity with an average day temperature of 23 °C and night temperature of 18 °C) where watering occurred every three days. The experiment was carried out twice and plant survival was recorded after two weeks of transplant.

### 4.2. Droplet-Vitrification Cryopreservation

#### 4.2.1. Plant Material

**Figure 9:**
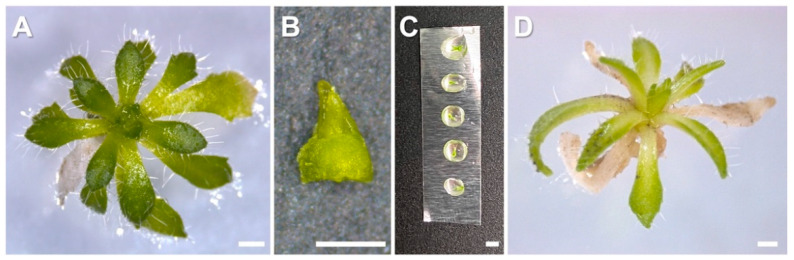
Main steps of droplet-vitrification cryopreservation procedure and recovery of Yukon Draba shoot tips. (**A**) A 2-week-old plant from which shoot tips were excised; (**B**) a shoot tip (1.0 mm) used for cryopreservation; (**C**) An aluminum foil strip with vitrification solution (PVS3) droplets containing shoot tips. (**D**) A normal shoot recovered from cryopreserved shoot tip after 2 weeks of culture. Bars = 1 mm.

In vitro shoots maintained on BM for 4 weeks were subcultured to shoot multiplication medium (SMM) consisting of BM supplemented with 2.2 μM BA and 1 μM GA_3_ and grown for a period of two weeks to promote shoot multiplication (Figure 9A). To determine the effect of stock plant age on the efficiency of cryopreservation, shoot tips were excised from the cultures after 1, 2, 3 and 4 weeks of sub-culture. Shoot tips (1 mm in length, Figure 9B) containing 5–6 leaf primordia (LPs,) were excised and maintained on SMM in Petri dishes and incubated for 24 h in darkness at 25 ± 2 °C. Shoot tips were transferred to liquid preculture BM with 0.3 M sucrose. Shoot tips were precultured for 1, 2, 3 and 4 days at 25 ± 2 °C in the dark and placed on a shaker (90 rpm, MaxQ 2000, Fisher Scientific, Ottawa, ON, Canada).

Precultured shoot tips were treated for 20 min with a loading solution (LS, liquid BM + 1.9 M glycerol + 0.5 M sucrose, pH 5.8) at room temperature on a shaker (90 rpm). Shoot tips were then transferred to plant vitrification solution 3 (PVS3; Nishizawa et al. 1993; liquid BM + 50% sucrose + 50% glycerol at pH 5.8) at room temperature for 20, 30, 40, 50, 60 min. PVS3-treated shoot tips were placed on single droplets of 2.5 μL PVS3 solution kept on an aluminum foil strip followed by direct immersion into liquid nitrogen for 1 h (Figure 9C).

After one hour of LN immersion, the aluminum foil strips with shoot tips were warmed quickly into unloading solution (ULS; liquid BM + 0.8 M sucrose at pH 5.8) for 60 s in the water bath set at 40 °C, and then at 25 ± 2 °C for additional 30 min. Thawed shoot tips were removed from ULS and cultured for recovery (postculture) on SMM for 1 day in the dark, for 3 days under low light (5 μmolm^−2^ s^−1^), and then moved to normal growth room condition as mentioned above. The survival rate was calculated three days after thawing and the regrowth rate was calculated 2 weeks after thawing (Figure 9D). All cryoprotectant solutions were sterilized by vacuum filtration through 0.2 μm filters (Nalgene™, ThermoFisher Scientific^®^, Burlington, ON, Canada). The shoot tips that became green after seven days were counted as survived and those that continued development into plantlets after 20 days were counted as the shoot tips showing regrowth.

#### 4.2.2. Thermal Analysis

The Phase transitions in shoot tips of Yukon Draba were studied using a Thermal Analysis Differential Scanning Calorimeter (DSC) (DSC1, Mettler Toledo, Leicester, UK), calibrated with zinc (422.81 °C, 115.57 Jg^−1^) and indium (156.85 °C, 29.03 Jg^−1^) standards. Samples were sealed in 40 μL aluminum pans, placed in the DSC at 22 °C, and then cooled at a rate of −10 °C min^−1^ to −80 °C, at which the sample was held isothermally for 5 min before rewarming to 25 °C at a rate of 10 °C min^−1^.

Samples tested included (i) control shoot tips without any treatment, (ii) shoot tips precultured in 0.3 M sucrose for 24 h, (iii) shoot tips precultured in 0.3 M sucrose for 24 h followed by loading solution for 20 min, (iv) shoot tips precultured in 0.3 M sucrose for 24 h followed by loading solution for 20 min, and finally PVS3 (20, 30, 40, 50 and 60 min). Each treatment was replicated twice. Peaks were analyzed using STARe thermal analysis software (Mettler Toledo, Leicester, UK).

### 4.3. Statistical Analysis

Data were analyzed using PROC GLIMMIX in SAS 9.4 software (SAS Institute Inc. Cary, NC, USA). The micropropagation experiments were arranged as a completely randomized design and analyzed with one-way ANOVA. Treatment levels were replicated ten times with four shoot tips in each replicate and the experiment was repeated three times. Normality was tested using Shapiro–Wilk’s test of normality. Means were compared using Tukey–Kramer Honest Significant Difference (HSD) test with an alpha value of 0.05. For cryopreservation experiments, there were ten samples per treatment with three replicates (*n* = 3) of shoot tips obtained from micropropagated cultures and the experiment was repeated twice. The data obtained under this experiment were analyzed by one-way ANOVA and Turkey’s range test using SPSS 24.0 version using a generalized linear mixed model. All data were presented as means ± standard errors and different letters in the tables and figures indicated significant differences at *P* = 0.05.

## 5. Conclusions

In summary, the protocol for micropropagation and cryopreservation of Yukon Draba was successfully developed from the seedling which served as the source for establishing shoot cultures. The shoot tip explants were multiplied in vitro with optimization of basal medium, sugar, and plant growth regulators and rooted plantlets were successfully transferred to the greenhouse. The shoot tips were efficiently cryopreserved with the droplet-vitrification procedure and plants were successfully regenerated from cryopreserved tissues. This study shows the wide application of tissue culture techniques for long term conservation of Yukon Draba and is the first report of successful micropropagation and cryopreservation of this species. As such, these findings may be useful for in vitro conservation, replenishment, and biodiversity maintenance of other endangered plant species.

## Data Availability

No new data were created or analyzed in this study. Data sharing is not applicable to this article.
